# Fly Ash as an Eco-Friendly Filler for Rigid Polyurethane Foams Modification

**DOI:** 10.3390/ma14216604

**Published:** 2021-11-02

**Authors:** Monika Kuźnia, Anna Magiera, Beata Zygmunt-Kowalska, Katarzyna Kaczorek-Chrobak, Kinga Pielichowska, Piotr Szatkowski, Aleksandra Benko, Magdalena Ziąbka, Wojciech Jerzak

**Affiliations:** 1 Department of Heat Engineering and Environment Protection, Faculty of Metals Engineering and Industrial Computer Science, AGH University of Science and Technology, Mickiewicza 30 Av., 30-059 Krakow, Poland; asocha@agh.edu.pl (A.M.); zygmunt@agh.edu.pl (B.Z.-K.); wjerzak@agh.edu.pl (W.J.); 2 Instytut Techniki Budowlanej, Filtrowa 1, 00-611 Warszawa, Poland; k.kaczorek-chrobak@itb.pl; 3 Department of Biomaterials and Composites, Faculty of Materials Science and Ceramics, AGH University of Science and Technology, Mickiewicza 30 Av., 30-059 Krakow, Poland; kingapie@agh.edu.pl (K.P.); pszatko@agh.edu.pl (P.S.); abenko@agh.edu.pl (A.B.); 4 Department of Ceramics and Refractories, Faculty of Materials Science and Ceramics, AGH University of Science and Technology, Mickiewicza 30 Av., 30-059 Krakow, Poland; ziabka@agh.edu.pl

**Keywords:** composite materials, eco-friendly fillers, fly ash, microspheres, rigid polyurethane foams

## Abstract

There is currently a growing demand for more effective thermal insulation materials with the best performance properties. This research paper presents the investigation results on the influence of two types of filler on the structure and properties of rigid polyurethane foam composites. Fly ash as a product of coal combustion in power plants and microspheres of 5, 10, 15, and 20 wt.%, were used as rigid polyurethane foams modifiers. The results of thermal analysis, mechanical properties testing, and cellular structure investigation performed for polyurethane composites show that the addition of fly ash, up to 10 wt.%, significantly improved the majority of the tested parameters. The use of up to 20 wt.% of microspheres improves the mechanical and thermal properties and thermal stability of rigid polyurethane foams.

## 1. Introduction

One of the most popular and commonly used thermosetting polymeric materials is rigid polyurethane (PUR) foam [1]. Due to its low thermal conductivity, it is mainly used in the building and construction industry [2]. Moreover, its good performance properties, i.e., low apparent density (25–50 kg/m^3^) [3], chemical resistance, good thermal and mechanical characteristics [4,5], resulted in the more widespread usage of PUR in other industries, including automotive, furniture for public transportation, astronautics, refrigerators and petrochemical engineering [6,7,8].

Since markets’ demands for PUR is considerable, growth in production of this material is observed every year. According to the Global Rigid Polyurethane Foam Sales Market Report 2021, published by Industry Research, the global market of this type of materials was valued at USD 6979 in 2020 and will reach USD 10,410 million by the end of 2027 [9]. The vast interest in thermal insulating materials, in particular PUR foam, mobilized researchers to seek new solutions when producing PUR materials. Two of the most recognizable motives are the willingness to reduce production costs and the tendency to eliminate petrochemical products from the environment. The introduction of the fillers can modify PUR during the production process. Numerous studies focused on the filler addition effect on the physical properties of PUR foams, such as density, thermal and mechanical properties, polyurethane cell structure and flame retardancy [10,11,12,13,14,15,16,17]. Kuźnia et al. [18] modified PUR foam with fluidized bed combustion fly ash, a waste material rarely used in the industry. With up to 20 wt.% of the filler, foam showed improved thermal stability, reduced carbon contents, and gross calorific values. Choe et al. [19] used chemically treated CaCO_3_ fillers to improve the acoustics and mechanical properties of the foams. The research obtained composite materials with lower porosity and higher compressive strength than not chemically modified fillers.

In this study, PUR composite was prepared using two types of fillers: fly ash (FA) from a pulverized bituminous coal-fired boiler and microspheres (M), which were the separated fraction of FA. FA generated in pulverized boilers was used as source material in geopolymers [20,21] and concrete [22], as well as thermal properties’ modifier in polymer composites [23,24,25] and flame-retardant in low-density polyethene [26]. M represent valued performance properties, including low density, thermal and mechanical stability, hydrophobicity. They were used as a component of insulating and non-flammable materials, composites and light concrete [23,26,27,28,29,30]. Thus, FA and M consist of a cheap, potential filler and flame-retardant in PUR production.

Furthermore, both species are waste materials and using them in the PUR formulation process can diametrically can significantly reduce the negative impact on the environment resulting from their storage. The authors attempt to describe the influence of the aforementioned fillers and their concentration on PUR physical and performance properties. PUR composite characteristics, which were under investigation, were: polyurethane cellular structure, chemical structure, mechanical properties, thermal properties and thermal stability. The most frequently studied fillers used in PUR materials are mainly flame-retardant additives. There are a few publications that related to the use of FA and M in the PUR modifications [18,31,32]. Therefore, the research topic discussed in this article is innovative and there are no published research results in this field.

The addition of M and FA to other polymeric materials is used in polymer chemistry, i.e., FA-reinforced thermoplastic starch composites [33] or other polymer composites reinforced with FA [34,35,36]. The most frequently studied fillers used in PUR materials are mainly flame-retardant additives. There are a few publications that related to the use of industrial waste in the PUR modifications. Therefore, the research topic discussed in this article is innovative and there are no published research results in this field. The latest research has shown that FA might express high levels of toxicity on human health [37]. The research was assessed in vitro using HeLa cells and Jurkat cells. The size of ash particles appeared to be an important determinant of their toxicity. Authors realize that further research is needed to evaluate the toxicity of polyurethane materials doped with FA prior to practical used.

## 2. Materials and Methods

### 2.1. Characterization of the Fly Ash and Microsphere

**Figure 1:**
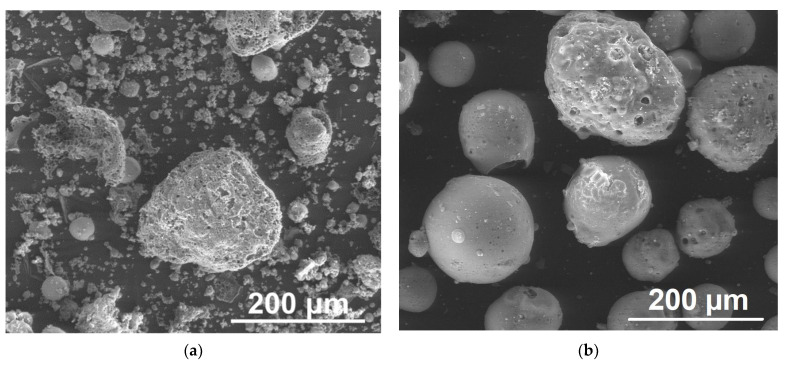
SEM images of FA (**a**) and M (**b**). Magnitudes ×350.

FA, obtained from one of the Polish power plants and generated in a pulverized bituminous coal-fired boiler, and M, acquired from one of Kazakh coal-fuelled power plant and separated from FA by flotation method in were taken for an investigation. Kazakhstan’s M are characterized by a larger grain diameter and a higher Al_2_O_3_ content compared to Polish. FA and M were used for formulation in unmodified form. Scanning electron microscope (SEM) images (Figure 1) illustrate the shape of FA, and M. FA particles were rounded, and coarse, sparse M could be detected. 

**Figure 2:**
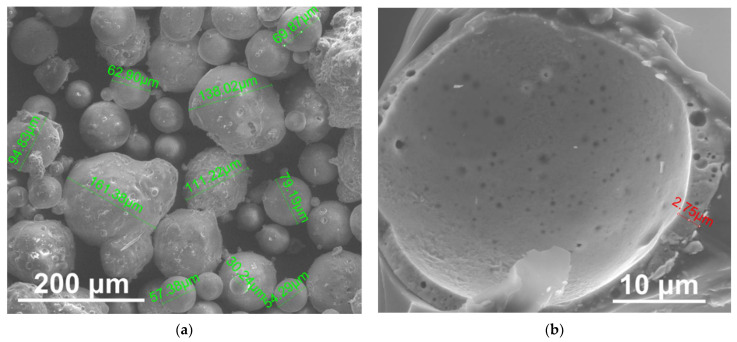
SEM images of different sizes of M (**a**) and broken M (**b**). Magnitudes×350 (**a**), and ×5000 (**b**).

In most cases, M constituted about 1–2% of FA obtained from coal combustion. M are filled with exhaust gases, which are present in the coal boiler; mainly with CO_2_ and N_2_. They consist of a spherical structure. The diameter of grains was smaller than 200 μm, while in most cases, their dimensions ranged from 1 to 150 μm (Figure 2a), and the shell thickness was several micrometers (Figure 2b). M were mainly cenospheres, and their surface was coarse.

In the primary stage of this research, FA and M composition and morphology were characterized. The detailed results of these studies were published elsewhere [30,31]. The median particle size (D50) of FA, measured by the particle size analyzer (Malvern Mastersizer 2000 Ver. 5.60; Malvern Instruments Ltd., Malvern, UK), was 159 μm. The chemical composition was analyzed by an X-ray fluorescence (XRF) spectrometer. Chemical composition of FA and M is shown in Table 1. FA has been found to consist of SiO_2_ (52.3%), Al_2_O_3_ (27.4%), Fe_2_O_3_ (7.9%), K_2_O (4.2%), CaO (3.0%), TiO_2_ (1.4%), and SO_3_ (1.2%). Mineral phases, identified and quantified using the X-ray diffraction (XRD) method, were mullite and quartz. The physical density of FA was 2.15 g/cm^3^. The median particle size (D50) of M was 158 μm. M were composed of SiO_2_ (52.8%), Al_2_O_3_ (39.9%), CaO (1.4%), Fe_2_O_3_ (1.4%), TiO_2_ (1.2%) and P_2_O_5_ (1.0%). M was mainly amorphous, and mineral phases of mullite, corundum and quartz were detected. Physical density was 0.39 g/cm^3^. 

### 2.2. PUR Foams Formulation

Polyurethane (PUR) rigid foams were prepared using the two-component commercial system EKOPRODUR PM4032 (PCC Rokita S.A., Brzeg Dolny, Poland). Both components, polyol and isocyanate (weight ratio 100:120), were mixed using a mechanical stirrer (4500 rpm for 1 min) and cast into rectangular mold dimensions of 20 × 20 × 5 cm, then left under a fume hood for the polymerization reaction to terminate. After 48 h of setting, foams were removed from the molds and left under the fume hood for a further 5 days (to eliminate the unreacted isocyanate component). At the first stage of formulation, the proportionate amounts (5, 10, 15, and 20 wt.%) of the filler (FA or M) were added to the polyol component and then mixed with isocyanate. The composite foam samples were labelled as PUR + FA5, PUR + FA10, PUR + FA15, PUR + FA20, PUR + M5, PUR + M10, PUR + M15 and PUR + M20.

### 2.3. PUR Foams Characteristics

The morphology of the cellular PUR structure was analyzed using the optical microscope Keyence VHX-900F (Keyence, Osaka, Japan). PUR foam samples were prepared by cutting into regular cuboids with dimensions 5 × 5 × 0.5 cm. Optical microphotographs were registered from different areas of each sample. Images were analyzed using the ImageJ (version 1.48v) free software. Horizontal and vertical Feret diameters characterizing the cellular PUR matrix structure were measured. Average values of diameters, together with standard deviations, were calculated from 50–60 counts. Other parameters measured were strut thickness (average and SD values of 50–60 counts) and a closed cells’ per cent. Additional observations were performed using the scanning electron microscope, SEM (Nova NanoSEM 200; FEI Company, Hillsboro, Oregon, USA). The cubic samples with dimensions 0.5 × 0.5 × 0.5 cm, then coated with gold and observed with the acceleration of 10 kV.

Fourier-transform infrared (FTIR) spectroscopy was used to define the molecular structure of foams. IR spectra were registered on Tensor 27 spectrometer (Bruker Optics, Billerica, Massachusetts, USA), operating with OPUS 7.2 software. Spectra were collected in the mid region of 4000–400 cm^−1^ after 64 scans at 4 cm^−1^ resolution in absorbance mode using the KBr pellet method. Materials analyzed were PUR foam, powdered FA and M, and composite foams PUR + FA20 and PUR + M20 (other composites were not included in testing due to low filler content).

Mechanical testing of the materials was performed on the universal testing machine Zwick 1435 (Zwick Roell, Ulm, Germany; load cell 5 kN). Foam samples were prepared in the form of cylinders, with diameters of 30 mm and to heights of 12 mm and compressed at a speed of 2 mm/min until 75% deformation. Compressive strength (R_s_) and Young’s modulus (E), were calculated from the obtained stress-strain curves. For each type of material, the results of the measurements were summarized as the average of six tests and the standard deviation (SD) value.

Thermal analysis was conducted using differential scanning calorimeter (DSC), Mettler Toledo DSC1 (Mettler Toledo, Switzerland) in the inert atmosphere of nitrogen. The 3–4 mg were closed in pierced aluminum pans and measured (heated/cooled) at the rate of 10° samples C/min in the range of −50–240 °C. Thermogravimetric analysis (TGA) was also carried out using Discovery TGA 550 instrument (TA Instruments, New Castle, DE, USA). The 5–6 mg samples were tested in platinum pans in the nitrogen atmosphere in the range of temperatures 45–700 °C at the heating rate of 10 °C/min.

## 3. Results and Discussion

### 3.1. Cellular Structure

**Figure 3:**
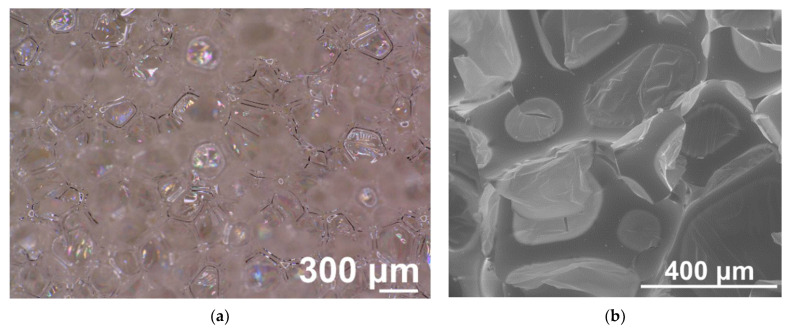
Optical (**a**) and SEM image **(b**) of the cross-sections of PUR foam. Magnitudes ×100 (**a**), and ×200 (**b**).

The reference unmodified PUR foam was also taken for investigation under the optical microphotograph (Figure 3a). The well-formed cellular structure can be seen on the cross-section of a reference sample. The observed cells were polyhedron in shape, uniform (SD values were small and did not exceed 8% of the average values), most of them were closed, which is an important characteristic influencing thermal insulation properties of the PUR foams [32,38,39]. 

Strut thickness (Table 2) of the cells was approximately 20 μm, vertical and horizontal Feret diameters were 193 and 207 μm. The similarity in both Feret diameters values indicated no favorable growth direction within the cross-sectional area of the sample. SEM image (Figure 3b) shows minor disruption of the cellular structure caused by the coating with the gold process.

**Figure 4:**
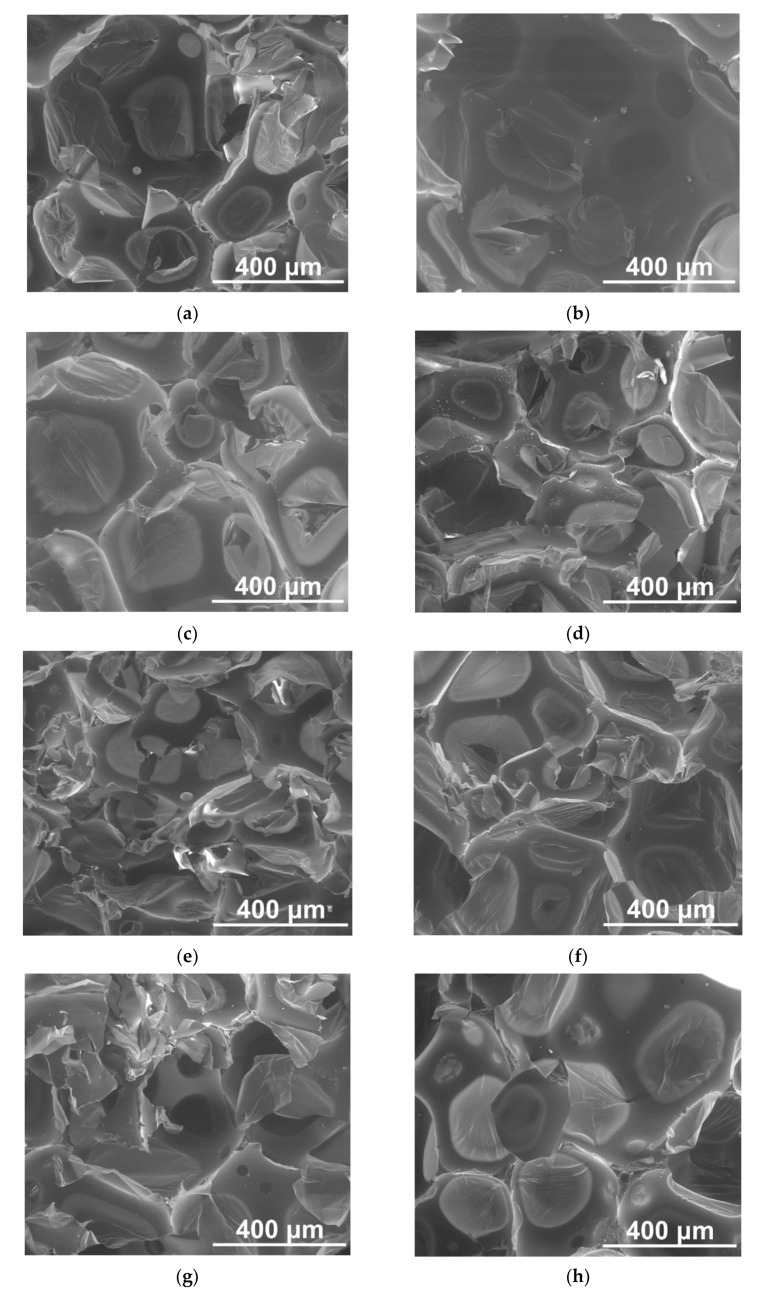
SEM images of the cross-sections of PUR foams modified with 5 (**a**,**b**), 10 (**c**,**d**), 15 (**e**,**f**), and 20 wt.% (**g**,**h**) of FA (left column) and M (right column). Magnitudes ×200.

SEM images of the cross-sections of PUR foams modified with FA and M are given in Figure 4. The performed analysis pointed out that the addition of both types of filler did not disrupt the cellular structure of the PUR matrix; however, a decrease in closed cells’ content was observed, reaching approx. 84%. 

Bigger particles of both FA and M impaired the PUR cellular structure, resulting in opening cell walls [22,40]. The polyhedron shape of the cells was observed. Fillers were evenly dispersed within a polymer matrix and placed between cells (Figure 4). A uniform cellular structure characterized all composite materials. The Feret diameter SD did not exceed 10% of the average values. Both cellular diameters were comparable, indicating no favorable growth direction. Strut thicknesses were approximately 20 μm and were not dependent on the composite composition. Incorporating both types of filler caused a decrease in vertical and horizontal Feret diameters, which suggested that FA particles and M acted as nucleation sites during the foam formation [22,39]. Moreover, an increase in viscosity of the PUR + FA and PUR + M mixtures and an increasing filler content hindered cell growth during foam formation [22,41]. 

### 3.2. Chemical Structure and Mechanical Properties

**Figure 5:**
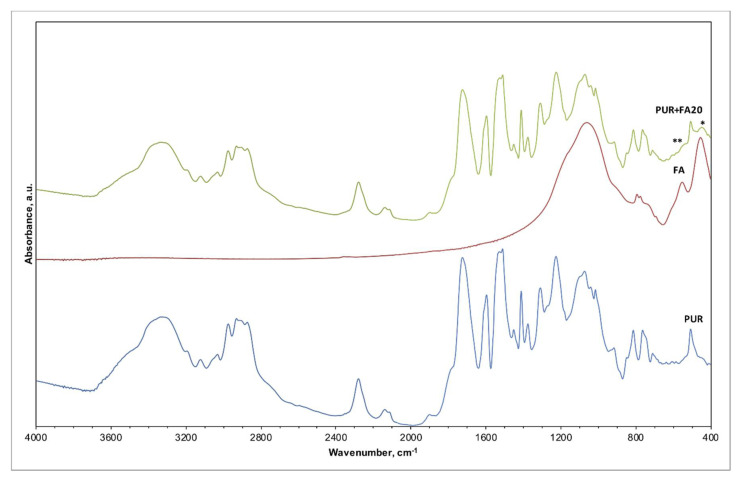
IR spectra of PUR foam, FA, and PUR foam modified with 20 wt.% of FA (PUR + FA20p).

**Figure 6:**
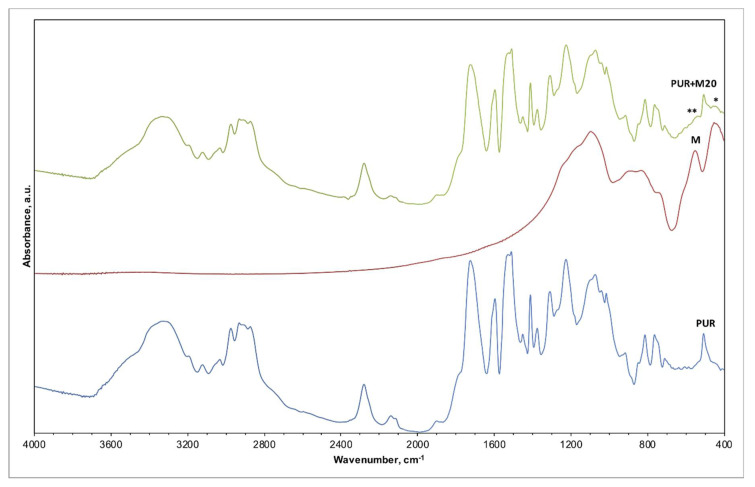
IR spectra of PUR foam, M, and PUR foam modified with 20 wt.% of M (PUR + M20p).

The IR spectra of pristine PUR foam, as received FA, and composite foam material PUR + FA20p were compared (Figure 5, Table 3)), as well as the analogical spectra of PUR + M20p (Figure 6, Table 4). Spectra registered for unmodified PUR foam are shown on the bottom of both plots. Based on XRD analysis, crystalline phases present in the FA sample were mullite and quartz [30]. M were mainly composed of an amorphous phase; mineral phases detected were mullite, corundum and quartz. The similarity in the crystalline composition resulted in comparable IR spectra registered for both FA and M. Bands associated with the silica (quartz), seen on the spectrum of FA (Figure 5; middle plot) and M (Figure 6; middle plot). 

The IR spectrum of PUR + FA20p (Figure 5; top plot) and PUR + M20p (Figure 6; top plot) overlapped with the spectrum of unmodified PUR foam due to the abundant quantity of polymer matrix in the composite. The majority of characteristic bands for both PUR and FA/M occurred in the same wavenumber ranges (Table 3), only slight changes in the spectrum of PUR + FA20p/PUR + M20p suggested the presence of the filler in the polymer matrix: (*) 550 cm^−1^ (aluminosilicate); and (**) 460 cm^−1^ (silica). No additional bands belonging to other chemical species were observed, indicating that there is no chemical bonding between polymer and filler.

The values of compressive strength (R_s_) and Young’s modulus (E), obtained during mechanical testing, are presented in Table 5. The incorporation of both fillers, up to 20 wt.%, increased both R_s_ and E, which suggested the interfacial interactions between polymer matrix and fillers and uniform distribution of fillers within PUR foams. The presence of M in composite foams resulted in better mechanical characteristics of the samples, which correlated with the conclusions of other research groups [48,49].

### 3.3. Thermal Properties

DSC analysis was performed in order to evaluate phase transitions within PUR materials during heating. The glass transition temperatures (T_g_) and changes in heat capacity (∆C_p_) are presented in Table 6. 

The T_g_ for unmodified PUR foam were approximately 43 and 150 °C. Incorporating both types of filler decreased these parameters, which suggested that they acted as plasticizers and decreased interactions between PUR chains. When the content of fillers increased T_g,1_ was still lower than for pristine PUR foam but higher than parameters calculated for 5 and 10 wt.% content of the filler. This phenomenon was caused by the lower mobility of polymer chains within the matrix structure resulting from the presence of filler particles. The value of ∆C_p,1_ for unmodified PUR foam was approximately 0.2 J g^−1^ K^−1^ and was the highest value of all samples analyzed, which was related to the greatest mobility of the polymer chains. When fillers were introduced into the PUR structure, changes in heat capacity declined. The values of ∆C_p,2_ for all materials were comparable (approximately 0.25 J g^−1^ K^−1^).

**Figure 7:**
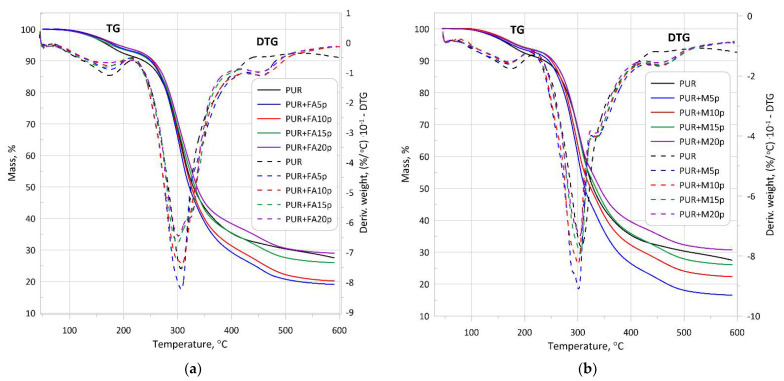
TGA and DTG curves of unmodified PUR foam and composite materials containing FA (**a**), and M (**b**).

Thermogravimetric analysis (TGA) was carried out under an anaerobic atmosphere in nitrogen to investigate the thermal degradation process of the PUR foam. Figure 7 shows TGA curves registered for the foams with 0, 5, 10, 15 and 20 wt.% of fillers. 

The corresponding thermal parameters are marked in Table 7. These were: T_5%_- which represented initial degradation temperature corresponding to 5% weight loss; T_10%_ and T_50%_-temperatures at 10 and 50% weight loss, respectively; T_1_ and T_2_-temperatures with maximum degradation rates in first and second degradation stage, respectively (as the DTG peaks maximum); and R_1_ and R_2_-the maximum rate of degradation (% °C^−1^) in first and second degradation stage, respectively.

The initial mass loss stage (T_5%_) is beginning at a temperature range of 170–190 °C. In the case of unmodified PUR foams, the 10 wt.% weight loss occurred at the lowest temperatures (238 °C) compared to composite materials (about 250 °C). The thermal degradation of all modified PUR foams showed a two-stage degradation. The first step (main degradation process) occurred in the temperature range of 220–450 °C when hard segments of the PUR structure were destroyed which involved dissociation of the polyol and isocyanate components. In this stage, the highest rate of weight loss (R_1_) appeared at temperature T_1_ (about 300 °C). The addition of 5 and 10 wt.% of filler (both FA and M) improved the thermal stability of modified foams. Lower temperatures (T_1_) of composites with 15 and 20 wt.% FA and unmodified PUR can be attributed to earlier chemical decomposition of PUR materials. Addition of filler in excessive amounts (15 and 20 wt.% of FA) destroy the polymer structure and contribute to earlier degradation. The largest residue after the thermal analysis at 590 °C occurred in the case of PUR with the addition of 20 wt.% filler and amounted to approx. 30% (for unmodified PUR is 28%). On the other hand, the smallest amount of residue was in the case of using the filler (FA and M) in the amount of 5%. The FA occurrence in the content of 15 and 20 wt.% impaired the cellular structure of PUR and had a slightly negative effect on its thermal resistance. However, the addition of M, even up 20 wt.%, did not adversely affect the thermal stability of the PUR structure. This might be due to the oval shape of the M, which did not damage the PUR cell walls, as shown in Section 2.1 in Figure 2. The second degradation step occurred in the temperature between 420 and 590 °C, and the maximum rate of weight loss (R_2_) occurred at temperature T_2_ equal to 450 °C. The second degradation step was related to the soft segment decomposition and was slower and much less visible than the degradation of the hard segments.

## 4. Conclusions

The analysis provided in this research article shows that the addition of coal FA up to 10 wt.% improved the composite’s mechanical and thermal properties. The use of FA in the amount greater than 10 wt.% caused damage to cell walls in a polymer matrix, which caused the worsening of mechanical and thermal properties of polyurethane composites [18]. Coal FA particles, except for a small number of M (1–2%), contained particles of irregular shapes cause damages to the walls of growing polymer cells.

In the case of M as a filler, no negative influence on the structure of polyurethane cells was observed. This may be due to their oval shape, and therefore there is little possibility of damaging the walls of the growing polyurethane matrix. 

The research results on mechanical properties show that incorporating both fillers, up to 10 wt.%, increased both compressive strength and Young’s modulus because of the interfacial interactions between polymer matrix and filler particles. The addition of M to composite foams resulted in better mechanical properties than those obtained for FA-containing foams. 

The use of fillers does not change the chemical structure of the polymer because no new bonds appeared in the FTIR spectra of the analyzed materials. 

Further studies are necessary in order to obtain the data for other functional properties, e.g., fire properties (heat, smoke, toxic combustion products), water absorption, thermal conductivity, dimensional stability, of FA and M modified rigid polyurethane foams. The extended analysis might be performed for the PUR foams with a more complex construction. 

It can be seen that the content of the microspheres varies depending on where the samples are taken. The best solution would be to use a fly ash as a filler with a high content of microspheres, because the separation of microspheres is associated with higher costs. A very good option may be the addition of separated microspheres to fly ash with a small content of microspheres. The authors also carry out the fly ash silanization which make possible to increase the content of microspheres (the article is under preparation).

## Figures and Tables

**Table 1:** Chemical composition of flay ash and microspheres.

Fly Ash				Microspheres			
Element	Conc., %	Oxide	Conc., %	Element	Conc., %	Oxide	Conc., %
Mg	0.658	MgO	1.464	O	49.30	Na_2_O	0.464
Al	18.10	Al_2_O_3_	27.38	Si	24.69	MgO	0.274
Si	35.53	SiO_2_	52.31	Al	21.12	Al_2_O_3_	39.91
P	0.748	P_2_O_5_	0.880	Ca	1.016	SiO_2_	52.83
S	1.035	SO_3_	1.201	Fe	0.984	P_2_O_5_	1.017
K	9.383	Cl	0.000	Ti	0.728	SO_3_	0.032
Ca	6.434	K_2_O	4.153	P	0.443	K2O	0.502
Ti	2.819	CaO	2.954	K	0.416	CaO	1.421
V	0.115	TiO_2_	1.409	Na	0.344	TiO_2_	1.215
Cr	0.076	V_2_O_5_	0.058	As	0.246	Cr_2_O_3_	0.006
Mn	0.224	Cr_2_O_3_	0.033	Ba	0.237	MnO	0.033
Fe	21.29	MnO	0.078	Mg	0.165	Fe_2_O_3_	1.407
Co	0.094	Fe_2_O_3_	7.916	Sr	0.101	CuO	0.010
Ni	0.058	Co_3_O_4_	0.030	F	0.076	ZnO	0.004
Cu	0.042	NiO	0.015	Zr	0.032	Ga_2_O_3_	0.005
Zn	0.083	CuO	0.011	Cl	0.029	As_2_O_3_	0.325
Ga	0.011	ZnO	0.022	Mn	0.026	Rb_2_O	0.002
As	0.004	Ga_2_O_3_	0.003	S	0.013	SrO	0.119
Br	0.004	GeO_2_	0.000	Cu	0.008	Y_2_O_3_	0.006
Rb	0.035	Br	0.001	Y	0.005	ZrO_2_	0.044
Sr	0.074	Rb_2_O	0.008	Cr	0.004	BaO	0.264
Y	0.006	SrO	0.018	Ga	0.003	PbO	0.002
Zr	0.032	Y_2_O_3_	0.002	Zn	0.003	F	0.076
Ag	0.065	ZrO_2_	0.009	Pb	0.002	Cl	0.030
Sn	0.003	Nb_2_O_5_	0.001	Rb	0.002	-	-
Ba	0.021	Ag_2_O	0.015	-	-	-	-
Pb	0.043	SnO_2_	0.001	-	-	-	-
-	-	BaO	0.006	-	-	-	-
-	-	HgO	0.009	-	-	-	-
-	-	As_2_O_3_	0.001	-	-	-	-

**Table 2:** PUR cellular morphology parameters.

Sample Name	Vertical Feret Diameter, µm	Horizontal Feret Diameter, µm	Strut Thickness, µm	Closed Cells’ Content, %
PUR	193 ± 15	207 ± 16	20 ± 1	88
PUR + FA5p	150 ± 6	156 ± 7	17 ± 1	82
PUR + FA10p	138 ± 8	147 ± 8	19 ± 1	77
PUR + FA15p	170 ± 12	148 ± 11	22 ± 1	78
PUR + FA20p	181 ± 11	170 ± 10	22 ± 1	78
PUR + M5p	154 ± 8	149 ± 7	20 ± 1	83
PUR + M10p	169 ± 12	173 ± 11	21 ± 1	77
PUR + M15p	196 ± 18	192 ± 20	20 ± 1	70
PUR + M20p	152 ± 11	158 ± 9	20 ± 1	76

**Table 3:** Absorption bands of the PUR structure [22,42,43,44,45].

Wavenumber, cm^−1^	Characteristic Vibration
3330 1730 1510–1530 1310 1230 2970, 2870–2930 1450, 1410, 1340 1090 770–920 1600, 2280	Stretching vibration of N–H groups Stretching vibration of C=O bonds Bending vibration of N–H groups Stretching vibration of C–N bonds Stretching vibration of C–O bonds Symmetric and asymmetric stretching vibrations of C–H bonds in CH_2_ groups in aliphatic chains and CH_3_ end groups Vibrations of methylene and methyl groups δ bonds between C and O (ether bonds; related to the polyol structure) Skeletal vibrations of C–C bonds and aromatic rings (related to the isocyanate structure) Phenyl ring vibration (related to the isocyanate structure)

**Table 4:** Absorption bands of the FA and M structure [46,47].

Wavenumber, cm^−1^	Characteristic Vibration
1060	Asymmetric stretching vibration of Si–O(Si)
800–770	Symmetric stretching vibration of Si–O–Si
460	Bending vibration of O–Si–O in silicate tetrahedra
550	Al in tetrahedral positions

**Table 5:** Mechanical properties of the PUR foams calculated from stress-strain curves.

Sample Name	Compressive Strength, kPa
PUR	191.6 ± 14.4
PUR + FA5p	210.9 ± 11.5
PUR + FA10p	196.5 ± 15.9
PUR + FA15p	201.3 ± 27.1
PUR + FA20p	243.5 ± 9.4
PUR + M5p	234.5 ± 11.3
PUR + M10p	236.1 ± 11.3
PUR + M15p	235.2 ± 15.2
PUR + M20p	235.7 ± 25.0

**Table 6:** Glass transition temperatures and changes in heat capacity, calculated from DSC curves, for the PUR foams.

Sample Name	T_g,1_, °C	ΔC_p,1_, J g^−1^ K^−1^	T_g,2_,°C	ΔC_p,2_, J g^−1^ K^−1^
PUR	43	0.19	150	0.26
PUR + FA5p	−26	0.05	160	0.22
PUR + FA10p	−22	0.06	126	0.26
PUR + FA15p	−3	0.01	113	0.13
PUR + FA20p	−12	0.02	103	0.25
PUR + M5p	−32	0.07	131	0.31
PUR + M10p	−46	0.01	128	0.24
PUR + M15p	−42	0.06	128	0.35
PUR + M20p	−33	0.08	147	0.15

**Table 7:** Characteristics of thermal degradation of the PUR foams.

Sample Name	T_5%_, °C	T_10%_, °C	T_50%_, °C	T_1_, °C	R_1_, % °C^−^^1^	T_2_, °C	R_2_, % °C^−1^	Residue at 590 °C, %
PUR	172	238	329	306	0.79	-	-	28
PUR + 5FA	183	251	322	307	0.81	457	0.11	19
PUR + 10FA	191	253	326	308	0.73	451	0.10	20
PUR + 15FA	178	249	332	298	0.66	447	0.09	26
PUR + 20FA	191	255	336	302	0.64	448	0.09	29
PUR + 5M	176	245	313	301	0.90	451	0.09	17
PUR + 10M	187	250	325	301	0.81	450	0.09	22
PUR + 15M	185	254	334	305	0.77	459	0.09	26
PUR + 20M	184	254	342	304	0.71	449	0.08	31
